# Thymoquinone Suppresses the Proliferation, Migration and Invasiveness through Regulating ROS, Autophagic Flux and miR-877-5p in Human Bladder Carcinoma Cells

**DOI:** 10.7150/ijbs.60401

**Published:** 2021-08-12

**Authors:** Xuejian Zhou, Feifan Wang, Hongshen Wu, Xianwu Chen, Yan Zhang, Juntao Lin, Yueshu Cai, Jiayong Xiang, Ning He, Zhenghui Hu, Xiaodong Jin

**Affiliations:** Department of Urology, The First Affiliated Hospital, Zhejiang University School of medicine, Hangzhou, PR China.

**Keywords:** Bladder carcinoma, Thymoquinone, Autophagic flux, Reactive oxygen specifics, miR-877-5p/PD-L1 axis, Epithelial mesenchymal transition

## Abstract

Bladder carcinoma is among the top 10 most frequently diagnosed cancer types in the world. As a phytochemical active metabolic, thymoquinone (TQ) is extracted from seeds of Nigella sativa, possessing various biological properties in a wide range of diseases. Moreover, the outstanding anti-cancer effect of TQ is attracting increasing attentions. In certain circumstances, moderate autophagy is regarded to facilitate the adaptation of malignant cells to different stressors. Conversely, closely linked with the mitochondrial membrane potential (MMP) loss, the upregulation of intracellular reactive oxygen species (ROS) is reported to activate the cell apoptosis in many cancer types. Furthermore, the vital effects of microRNAs in the pathological processes of cancer cells have also been confirmed by previous studies. The present research confirms that TQ restrains the viability, proliferation, migration and invasion through activating caspase-dependent apoptosis in bladder carcinoma cells, which is mediated by TQ induced ROS increase in bladder carcinoma cells. Furthermore, TQ is proved to block the fusion of autophagosomes and lysosomes, causing the accumulation of autophagosomes and subsequent cell apoptosis. In addition, TQ is also found to initiate the miR-877-5p/PD-L1 axis, which suppresses the epithelial mesenchymal transition (EMT) and invasion of bladder carcinoma cells. Taken together, TQ induces the apoptosis through upregulating ROS level and impairing autophagic flux, and inhibiting the EMT and cell invasion via activating the miR-877-5p/PD-L1 axis in bladder carcinoma cells.

## Introduction

Worldwide, bladder carcinoma (BC) ranks the 10^th^ in the most commonly occurring malignancy forms, with an approximate 549,000 newly confirmed cases and 200,000 cancer related deaths in 2018 alone 1, 2. In male population, BC ranks the 6^th^ most frequent malignancy and the 9^th^ primary cause of carcinoma related deaths 3. Therefore, male predominance is clearly observed in the epidermiology of BC, with a four times higher of incidence in men compared to women 4, 5. 90% of BC belongs to urothelial carcinoma, in which 75% cases proved a non-muscle invasive bladder carcinoma (NMIBC), which is restricted to the mucosa (Ta or CIS) or submucosa (stage T1), with the rest cases showing a muscle invasive bladder carcinoma (MIBC) or metastatic disease (≥T2) 6, 7. Transurethral resection of bladder tumor (TURBT) combined with the tailored intravesical therapy is recommended as the first choice of treatment for NMIBC, and the radical cystectomy (RC) with extended lymphadenectomy is recommended as the standard treatment for MIBC 3, 8. In terms of adjuvant therapy, despite the various choices of chemotherapeutic agents or immune checkpoint inhibitors (ICIs), BC presents a high rate of progression, recurrence and metastasis, leading to worse clinical outcomes 3, 9. Therefore, the development of novel and effect adjuvant agents remains in urgent need.

With the advantages in safety, validity and variation, phytochemicals have emerged as a novel generation of anti-neoplastic medicine and gained increasing attentions of researchers 10. Among the thousands of phytochemicals that have been found, thymoquinone (TQ; C_10_H_12_O_2_) exerts promising and prominent bioactive characteristics 11. TQ was first extracted from black seeds of Nigella sativa in 1963 12, which is an ancient kind of herbal medicine, widely used in Mediterranean, West Asia and India areas for the management of various ailments 11. Therefore, TQ has been found to possess a range of bioactive properties, including anti-inflammation, antidiabetic, analgesic, immunoregulatory, neuro-protective and anti-neoplastic effects 13-15. Among the diverse bioactivities, the potent anti-carcinoma effect of TQ has been proved in multiple cancers, such as cervical carcinoma, breast carcinoma, colon carcinoma, bone carcinoma, liver carcinoma, lung carcinoma, prostate carcinoma and pancreatic carcinoma 13, 16, 17. The specific mechanisms of TQ exerts its antineoplastic effect are also various in different cancer types 11. For examples, TQ can retard the tumor growth of breast carcinoma by suppressing the PI3K/AKT axis 18; studies have also shown that TQ suppresses the A549 lung tumor cells proliferation by elevating the p53 expression and Bax/Bcl-2 ratio, and triggering the caspase 9 and caspase 3 at the same time 19. However, the researches pertaining to the effect of TQ against bladder carcinoma remain inadequate.

Reduction-Oxidation chemical reaction represents a crucial bioactivity of almost all living cells, providing necessary energy and materials for life processes. And the partial reduction of oxygen leads to the generation of Reactive oxygen species (ROS), which includes hydroxyl radical (HO), superoxide anion (O_2_^-^) and the hydrogen peroxide (H_2_O_2_) 20. ROS can function as an important signaling molecule, mediating a myriad of bioactivities, on the other hand, ROS could interact with pivotal cellular targets, including lipid, protein and DNA, leading to serious damage to cellular homeostasis 21. Various researches demonstrated that ROS could facilitate the anti-neoplastic effect of numerous chemicals, including sodium butyrate 22, artesunate 23, ginsenoside, and so on 24. Nevertheless, the way TQ regulates the level of intracellular ROS varies among different tissue and cell types 13, and whether TQ can exert its anti-cancer effect via regulating the level of ROS is still unknown.

As a highly conserved cellular process observed in almost all eukaryotes, autophagy features with a double-membraned vesicle, termed as autophagosome, which can sequester senescent organelles, damaged proteins or invading microbes and deliver these superfluous materials into lysosomes to form autolysosomes for further degradation 25. Autophagy plays a double-edged effect for cellular homeostasis, presenting intricate interactions with cell death 25, 26. However, an increasing number of studies found that autophagy plays a pro-survival role in highly proliferative or malignant cancer cells 27. The recycled bio-materials in autophagy are degraded and released back to the cytoplasm, facilitating the cellular metabolism and energy balance, protecting cancer cells against the intrinsic or extrinsic stressors 27. Therefore, targeting the autophagic flux holds great potential for the therapy of cancer 28. For example, ginsenoside can suppress the growth of human neuroblastoma cells through inhibiting the autophagic flux 24. This research also explored whether TQ could restrain the malignancy of bladder carcinoma through regulating autophagy flux.

As a class of short non-coding RNAs with 18-24 nucleotides, microRNAs (miRNAs) are found in both plant and animal cells, playing crucial part in various cellular processes, cell proliferation and differentiation included 29. miRNAs can recognize and bind to the target mRNAs according to sequence complementarity principle, resulting into the degradation or translation suppression of target mRNA 30. Furthermore, cancer formation and progression are results of deregulations of genes controlling cell differentiation and proliferation, where numerous studies have proved miRNAs play crucial and multifunctional effects 31. Therefore, regulating the level of specific miRNA or miRNA cluster has become a promising method to combat cancer. TQ has been shown the ability to regulate certain miRNAs in different tissue cells or disease models. Bashir AO et al. proved that TQ could potentiate the expression of miR-375 and miR-16 in hepatocellular carcinoma 32; and Bhattacharya S et al. proved that TQ administration through certain novel nanoparticles could suppress triple-negative breast cancer through modulating the expression level of miR-361 33. Accordingly, in this research, we also exploited the underlying mechanism of TQ inhibits the bladder carcinoma malignancy via regulating miRNAs.

PD-L1 (B7-H1 or CD274) plays an inhibitory role in activation of T cells by integration with PD-1, contributing T cell anergy and cancer cell immune evasion 34, 35. PD-L1 is upregulated in a series of human malignancies 35, and is associated with worse clinical outcomes. Besides, epithelial mesenchymal transformation (EMT) is a biological procedure via which epithelial cells lost its defining characteristics and acquire mesenchymal phenotype. EMT exerts indispensable effect in the process of cancer cells acquiring advanced ability of migration and invasion, featured with the downregulation of E-cadherin (E-cad) protein and upregulation of N-cadherin (N-cad) and vimentin proteins 36. Furthermore, there is a close connection between PD-L1 and EMT process. Abdullah Alsuliman et al. proved that over-expression of PD-L1 can promote the EMT process in claudin-low breast cancer 34, and similar results were also demonstrated by Satoshi Tsutsumi et al. in esophageal cancer cells 37. However, whether TQ can regulate these two biological markers in BC cells remains unclear.

## Materials and methods

### BC cells culture

BC cells (5637 and T24 cells) were obtained from Chinese Academy of Sciences. RPMI 1640 (Hyclone) was used for BC cell culture, added with 1% penicillin/streptomycin (Biological Industries) and 10% fetal bovine serum (FBS, Giboco), in 5% CO_2_ at 37 °C.

### Reagents and antibodies

Thymoquinone (TQ) was product of MCE (CAS: 490-91-5). Hoechst 33342 (B2261) was bought from Sigma-Aldrich. Cell counting kit‐8 (CCK‐8) was product of Selleck Chemicals (US). N-Acetyl-L-cysteine (NAC) and Z‐VAD‐FMK (ZVF) were obtained from Beyotime Biotechnology (Shanghai, China). The Apoptosis Detection Kit #556547 was purchased from BD (San Jose, CA). Antibodies against Bax (ab32503), Bcl‐2 (ab182858), LC3B (ab192890), Beclin-1 (ab207612), ATG7 (ab133528), MMP2 (ab92536), MMP9 (ab76003) and PD-L1 (ab213524) were bought from Abcam (San Francisco, CA). Antibody against vimentin (V6389) was purchased from Sigma-Aldrich (US). Antibodies against β‐actin (20536‐1‐AP) and Bcl-xl (10783-1-AP) were obtained from Proteintech (Chicago, IL). Antibodies against cleaved caspase 3 (9664), cleaved poly (ADP‐ribose) polymerase (PARP) (5625), E-cadherin (3195), N-cadherin (13116), and SQSTM1/p62 (8025) were obtained from CST company (NJ, US).

### Cell viability test

BC Cells (5637 and T24 cell lines) were seeded into 96 microplates (1×10^4^/well), added with 100 μL 1640 medium (supplemented with 10% FBS). After pre-administration of different agents for 24 h, 10 μL of CCK-8 reagent was instilled into each well. After 3 h of incubation at 37 °C, the absorbance (at 450 nm) was determined with a microplate reader.

### Transwell assay

A 24 well-chamber (costar, Corning, US) was used in transwell migration experiment. 5×10^4^ cells, suspended in RPMI 1640 medium (no FBS), was added in the apical chamber, while the basolateral chamber containing 1 ml complete RPMI 1640 medium (10% FBS) served as chemo-attractant. TQ of different concentration (0 µM, 1 µM and 2 µM) was administrated in each well for 24 h at 37 °C, then cells in the apical chamber was removed. In lower chamber cavity, migrated cells was fixed using 4% paraformaldehyde for 30 minutes, then immersed in crystal violet solution for 10 minutes. Cells were photographed with a microscope (Nikon, Tokyo, Japan) at 200× objective.

Matrigel (Becton Dickinson [BD], MA, US) was used for the transwell invasion assay to precoat the apical chamber. Cell suspicion containing 8×10^4^ cells was added into the apical chamber, and the following assay was conducted similarly to the above procedure.

### Colony formation assay

As for colony formation assay, 1×10^3^ cells were added into a culture dish of 4.5 cm^2^ in complete 1640 medium at 37 °C for 24 h. After that, fresh medium with TQ of different concentration (0 µM, 25 µM and 50 µM) was used for the culture of cells. The medium was changed every two days. After 10 days of grown, PBS solution was used to wash the cell colonies (cell counting >50) for once. 4% paraformaldehyde solution was used for the further fixation for 30 minutes, then cell colonies were immersed in 0.01% crystal violet solution for 10 minutes. Cell colonies were photographed by a researcher who was blinded to the grouping details of the research.

### Cell scratch test

Cancer cells were cultured till confluence. A vertical scratch wound was created in the culture dish using a 200 ul pipette tip, with nonadherent cells washed out with PBS solution. Then cells were incubated with 1640 medium (0.1% FBS) for 48 h, added with TQ (0 µM, 1 µM and 2 µM). The scratched wound was photographed at 0 h and 48 h with a microscopy (Nikon, Japan) using 100× objective. Healing rate of the wound was calculated by ImageJ software.

### Hoechst 33342 staining

BC Cells treated with TQ (50 µM, 24 h) or not were stained using Hoechst 33342 (1:1000) for 15 minutes. And then PBS was used to wash the residual dye twice. The morphological alterations of cell nucleus were photographed using a fluorescence microscopy.

### Cell apoptosis experiment

BC Cells were grown in a culturing dish of 9.6 cm^2^ to 90% of confluence. After pretreatment of various agents, cells were detached with trypsin and harvested by centrifugation at 1000 rpm for 3 minutes, then washed using PBS solution for 3 times and resuspended in 500 µl 1× binding buffer. Cell suspension was added with propidium iodide (PI, 5 µl) and Fluorescein isothiocyanate (FITC, 5 µl), respectively. Following incubation of 30 minutes away from light, cells were then analysed using flow cytometry (Becton Dickinson, CA).

### Western blot (WB) experiment

BC Cells were lysed using ice-cold RIPA (Applygen, Beijing, China) solution containing 1 mM phenylmethylsulfonyl fluoride (PMSF). Total protein was obtained by centrifugation, and the protein concentration was measured using BCA kit (Beyotime, China) according to manufacturer's stipulations. After addition of loading buffer and denaturation, an equal amount of total protein (20 ug) was resolved using SDS-PAGE then electroblotted onto PVDF membranes (Millipore, US) and blocked with nonfat milk (5%) for 1 h at RT. Next, the membranes were probed with relevant primary antibodies for 12 h at 4 °C, then washed with TBST solution. Secondary antibodies (1:5,000) were co-incubated with the membranes for 1 h at RT, and the band signals were detected using chemiluminescence (ECL) assay and Bio-Rad CD Touch Detection System.

### Transmission electronical microscopy (TEM)

Cells pretreated with TQ (50 µM) or not were harvested and fixed in glutaraldehyde (2.5%) for overnight. After washed for three times using PBS solution, cells were fixed in osmium tetroxide (1%) for 1 h, washed again for three times using PBS solution. An ascending grade of ethanol was used for the dehydration of cell samples, which were then embedded into spur resin for overnight. Then the samples were sectioned (thickness 50-60 nm) and stained with uranyl acetate and lead citrate. The subcellular structures were observed with a TEM (HITACHI, Tokyo, Japan).

### Intracellular ROS generation detection

ROS generation was determined using the ROS Assay Kit (Beyotime, Shanghai, China). An amount of 1×10^6^ cells were pretreated with different agents, harvested with trypsin and resuspended in 1 ml serum-free 1640 medium. Afterwards, the cells were incubated with 10 µM DCFH-DA for 0.5 h at 37 °C away from light, and washed with 1640 medium (no serum) for three times. Intracellular ROS level was analysed via flow cytometry.

### Mitochondrial membrane potential (MMP) test

JC-1 assay (Beyotime, Shanghai, China) was performed to determine the MMP changes of cancer cells. TQ of different concentration (0 µM, 25 µM and 50 µM) was pretreated to cancer cells, which were then harvested and then incubated in the JC-1 staining solution for 0.5 h at 37 °C away from light. Samples were suspended and washed using 1× JC-1 buffer solution twice and analysed using flow cytometry.

### Confocal microscopy observation

RFP-GFP-LC3B double fluorescence lentivirus (GENE, Shanghai, China) was used to transfect cancer cells at the 30% of confluence according to the manufacturer' protocol, and puromycin was used to obtain the stable transfectant of both cancer cell lines. After treated with indicated conditions, autophagosomes or autolysosomes were presented with intracellular yellow or red puncta, which were observed by an Olympus confocal microscope (Japan) and photographed via FLUOVIEW (Olympus) software.

### MicroRNAs

The inhibitor and mimic of hsa-miR-877-5p were purchased from TranSheepBio (Shanghai, China).

### Overexpression of PD-L1

Lentivirus carrying the transcript of CD274 (OBiO, Shanghai, China) was used for the transfection of 5637 and T24 cells strictly according to the instructions of the product. Following the transfection, puromycin was used for further selection of stable transfectant.

### Quantitative polymerase chain reaction (qPCR)

Total RNA extraction was conducted using NucleoZol (MACHEREY-NAGEL, Germany) reagent following the product protocol. The Takara reagent (CAT: RR047A) was used to synthesize complementary DNA (cDNA), and SYBR Green Master Mix (Yeasen, CAT:11201ES08) assay was conducted for the PD-L1 mRNA level analysis. Bio-Rad CFX96Touch PCR system (US) was used for the qPCR experiment. GAPDH was adopted as a control group, and the primer sequences were as below: GAPDH, Forward: GATATTGTTGCCATCAATGAC, Reverse: TTGATTTTGGAGGGATCTCG; PD-L1: Forward: CAATGTGACCAGCACACTGAGAA, Reverse: GGCATAATAAGATGGCTCCCAGAA.

The expression levels of various microRNAs were detected with Bio-Rad CFX96Touch PCR system (US) following the instructions of All-in-One miRNA qRT-PCR detection kit (US). Human U6 microRNA was chosen as the endogenous control. The difference in expression level of microRNA was described according to 2^-ΔΔCt^ calculation method. And the primer sequences used in this research were exhibited as below: hsa-miR-126-5p: CATTATTACTTTTGGTACGCG; hsa-miR-1277-5p: AAATATATATATATATGTACGTAT; hsa-miR-1286: TGCAGGACCAAGATGAGCCCT; hsa-miR-1292-5p: TGGGAACGGGTTCCGGCAGACGCTG; hsa-miR-183-3p: GTGAATTACCGAAGGGCCATAA; hsa-miR-194-3p: CCAGTGGGGCTGCTGTTATCTG; hsa-miR-32-3p: CAATTTAGTGTGTGTGATATTT; hsa-miR-335-3p: TTTTTCATTATTGCTCCTGACC; hsa-miR-573: CTGAAGTGATGTGTAACTGATCAG; hsa-miR-627-3p: TCTTTTCTTTGAGACTCACT; hsa-miR-7974: AGGCTGTGATGCTCTCCTGAGCCC; hsa-miR-876-3p: TGGTGGTTTACAAAGTAATTCA; hsa-miR-877-5p: GTAGAGGAGATGGCGCAGGG; hsa-miR-92b-3p: TATTGCACTCGTCCCGGCCTCC; hsa-miR-940: AAGGCAGGGCCCCCGCTCCCC.

### Animal models

Tumor xenograft models were established using male nude mice (Laboratory Animal Center, Zhejiang) of eight-week-old, which were raised in SPF environment with food and water ad libitum. T24 cells (2×10^6^) were suspended in 100 ul chilled PBS solutions and hypodermal inoculated in the flank of nude mice. Tumor volumes were documented and calculated every third day using a caliper, according to the formula: V=0.5×L×W^2^ (V, volume; L, length; W, width). After 10 days, the mice were casually assigned into 3 groups, which include control group (received normal saline (NS), i.p./every three days, n=4), TQ group (received TQ 15 mg/kg, i.p./every three days, n=4) and cisplatin (DDP) group (received 2.5 mg/kg, i.p./every three days, n=4). After 10 times of administration of NS, TQ, or DPP, mice were sacrificed and tumors were harvested, weighed and fixed in formalin solution (10%) for further immunohistochemical experiment and HE staining.

The lung metastatic model was performed using tail vein injection method. 8 nude mice were randomly divided into two groups, including control group (received NS, i.p./every three days, n=4) and TQ group (received TQ 15 mg/kg, i.p./every three days, n=4). After two administrations, T24 cells (1×10^6^) was injected to the tail veins of the nude mice in both groups, and the administration was continued for another 40 days. Then the mice were sacrificed and the lung tissues were obtained for further observation and HE staining.

All experiments acquired authorization of the Animal Care and Use Committee of The First Affiliated Hospital, School of Medicine of Zhejiang University. Meantime, all procedures were performed strictly following the stipulations of laboratory animals.

### Statistical analysis

ALL trials were conducted independently at least for three times, with all data documented and shown as mean ± standard deviation (SD). SPSS (23.0, IBM) was used to evaluate the variations of different groups with one-way or two-way analysis of variance. Statistical significance is confirmed when P value is less than 0.05.

## Results

### TQ inhibits the proliferation, migration and invasion of BC cells

**Figure 1:**
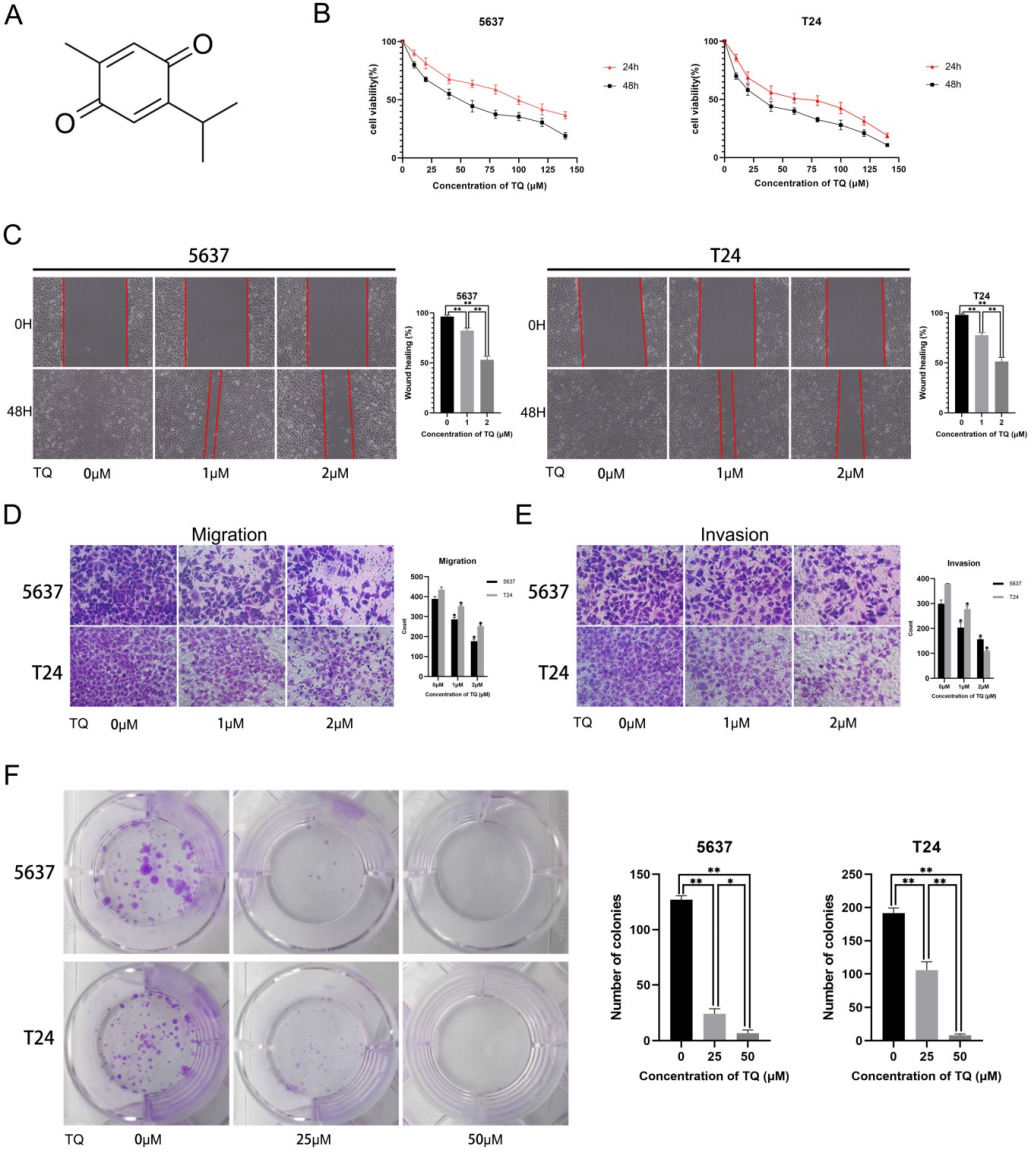
Thymoquinone (TQ) restrains the cell viability, migration, invasion and proliferation of bladder carcinoma (BC). (**A**) The molecular structure of TQ. (**B**) CCK-8 assay was conducted to test the viability of BC cells after TQ treatment of gradient concentration for 24 h and 48 h. Cell scratch test (**C**), transwell migration (**D**) and invasion (**E**) experiments were performed to detect the migration and invasion ability alteration of BC cells after administration of TQ (0 μM, 25 μM and 50 μM) for 24 h. (**F**) The colony formation assay of 5637 and T24 cancer cells with treatment of TQ (0 μM, 25 μM and 50 μM). The values of three independent replicate assays were presented as the mean ± standard deviation (SD). *p<0.05, **p<0.01.

The molecular structure of TQ is presented in Figure 1A. To explore the cytotoxic effect of TQ on BC cells, CCK8 assay was performed in both cell lines, with the result showing that TQ pretreatment significantly decreased the cell viability in time and dose dependent manners (Figure 1B). Furthermore, the IC50 values for the 24 h treatment of TQ were 75 µM and 60 µM in 5637 and T24 cell lines, respectively. As for the effect of TQ on migration ability of cancer cells, a cell scratch test (Figure 1C) and transwell migration assay (Figure 1D) were carried out, presenting that TQ dose-dependently suppressed the migration of both 5637 and T24 cells. Similarly, a transwell invasion assay (Figure 1E) presented that the invasion ability was also inhibited by TQ in a concentration dependent manner. Besides, the colony formation assay indicated that the proliferative ability of 5637 and T24 cells was dose-dependently refrained by TQ (Figure 1F), which is in accordance with the result of CCK8 assay. Altogether, these experiments conclude that TQ inhibits the proliferation, migration and invasion of both 5637 and T24 cancer cells.

### TQ induces caspase-dependent apoptosis in BC cells

**Figure 2:**
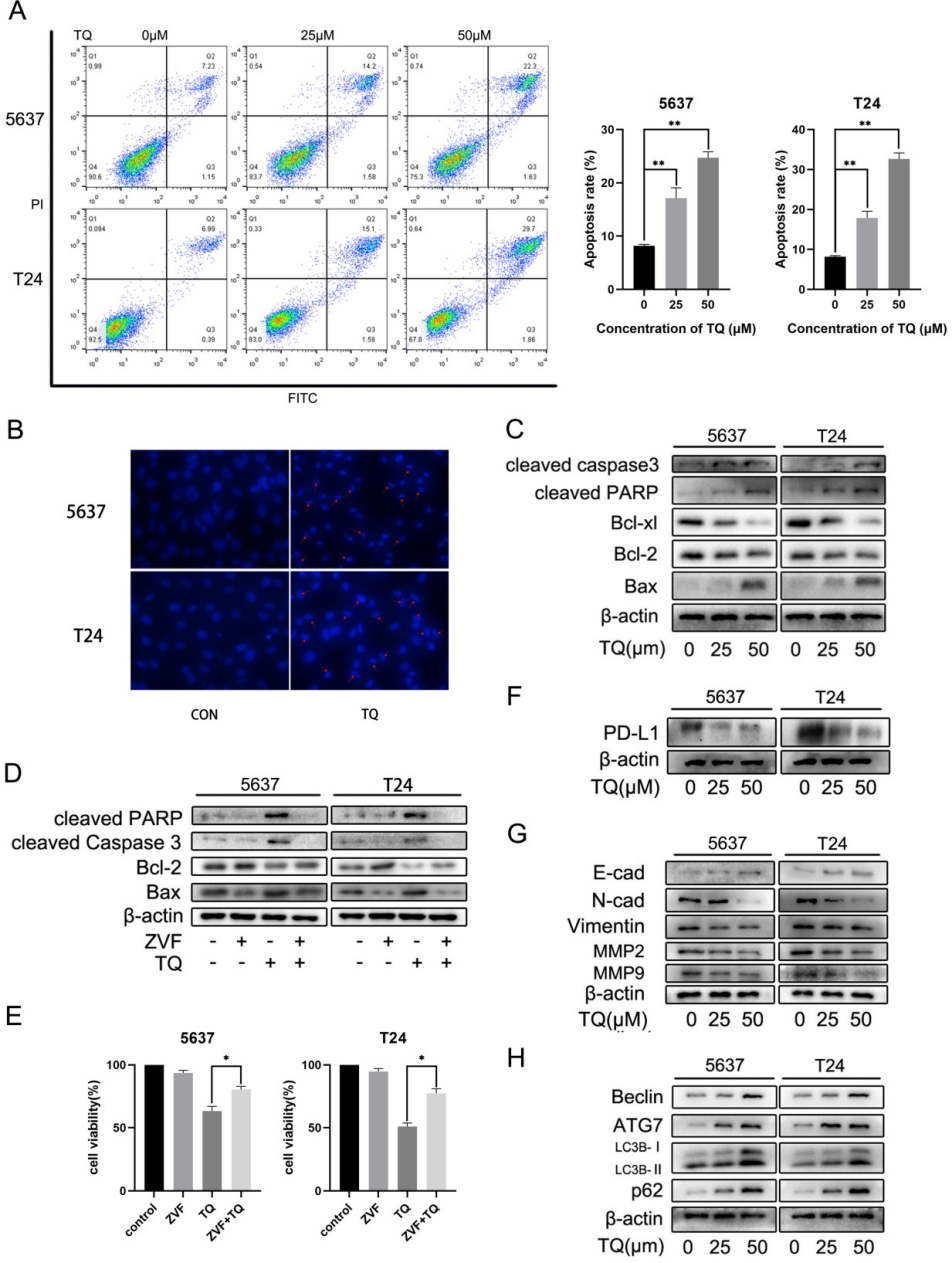
TQ initiates caspase dependent apoptosis in BC cells, as well as PD-L1 decrease and suppressed epithelial mesenchymal transformation (EMT). (**A**) Flow cytometry was conducted to test the apoptosis rate of BC cells after TQ treatment (0 μM, 25 μM and 50 μM) for 24 h. (**B**) Hoechst staining of BC cells after TQ treatment (50 μM, 24 h), red arrow head indicating the apoptotic cells. (**C**) WB assay showing the TQ induced changes of apoptosis related proteins, including cleaved caspase‐3, Bax, cleaved poly (ADP‐ribose) polymerase (PARP), Bcl-xl and Bcl‐2, with β-actin protein as endogenic control. After BC cells were treated with TQ (50 μM, 24 h) with or without the pretreatment of ZVF (20 μM, 4 h), WB (**D**) and CCK-8 assays (**E**) were conducted to assess the changes in apoptosis related proteins and cell viability, respectively. Following TQ treatment (0 μM, 25 μM and 50 μM) of 24 h, WB assays were also performed to detect the relevant protein changes of PD-L1 (**F**), cell invasion, EMT process (**G**) and autophagic course (**H**). Data of quantification were shown as the mean ± standard deviation (SD). ZVF: Z-VAD-FMK; WB: western blot; *p<0.05, **p<0.01.

To explore the underlying mechanism of TQ exerting its anti-tumor property in BC, we performed the flow cytometry assay (Figure 2A). FITC and PI were used in double staining of 5637 and T24 cells after TQ pretreatment for 24 h and the results implied that TQ could dose-dependently induce apoptosis in these two cell lines. Hoechst 33342 assay was conducted to stain the nuclei of cancer cells after TQ pretreatment (50 µM, 24 h) or not (Figure 2B). Because of the membrane permeability of Hoechst 33342, all the nuclei were stained as blue in the control group, while the apoptosis cell presented brighter, shrinked or fractured nucleus after TQ pretreatment, confirming the pro-apoptosis effect of TQ. Furthermore, WB assay also proved that TQ could increase the level of pro-apoptosis proteins in a concentration dependent manner, including cleaved caspase 3, cleaved poly (ADP‐ribose) polymerase (PARP) and Bax. However, the anti-apoptosis proteins, such as Bcl-2 and Bcl-xl, were downregulated as the concentration of TQ administration increased (Figure 2C). Moreover, as a cleaved caspase 3 inhibitor, the co-treatment of Z-VAD-FMK (ZVF) with TQ significantly reversed the TQ induced increase of cleaved caspase3 and cleaved PARP proteins (Figure 2D), notably rescued the cancer cells viability (Figure 2E), and markedly reduced TQ evoked cell apoptosis (Figure 5F).

### TQ decreases the PD-L1 expression, suppresses EMT progress and the invasion related proteins of BC cells

Apart from the central role in immune escape, PD-L1 protein also plays an essential effect in the EMT process and invasion of multiple malignancies. WB experiment was carried out to investigate the effect of TQ on the expression of PD-L1 protein. As is presented in Figure 2F, PD-L1 was detected in both 5637 and T24 cancer cells and was down-regulated by TQ in a dose-dependent manner. Besides, the EMT progress was markedly suppressed with the increase of treatment concentration of TQ (0 µM, 25 µM and 50 µM), represented with the enhancement of E-cad protein and decrease of N-cad and Vimentin proteins (Figure 2G). Furthermore, TQ also dose-dependently inhibited the invasion related proteins, demonstrated as lowering the expression of MMP2 and MMP9, which are crucial for the invasion ability of malignant cells (Figure 2G). Notably, the autophagy related biological markers were also found to be affected by TQ in a dose-dependent way (Figure 2H), which would be detailed discussed in part **3.6**.

### TQ impairs mitochondrial membrane potential (MMP) via upregulating intracellular ROS level in 5637 and T24 BC cells

**Figure 3:**
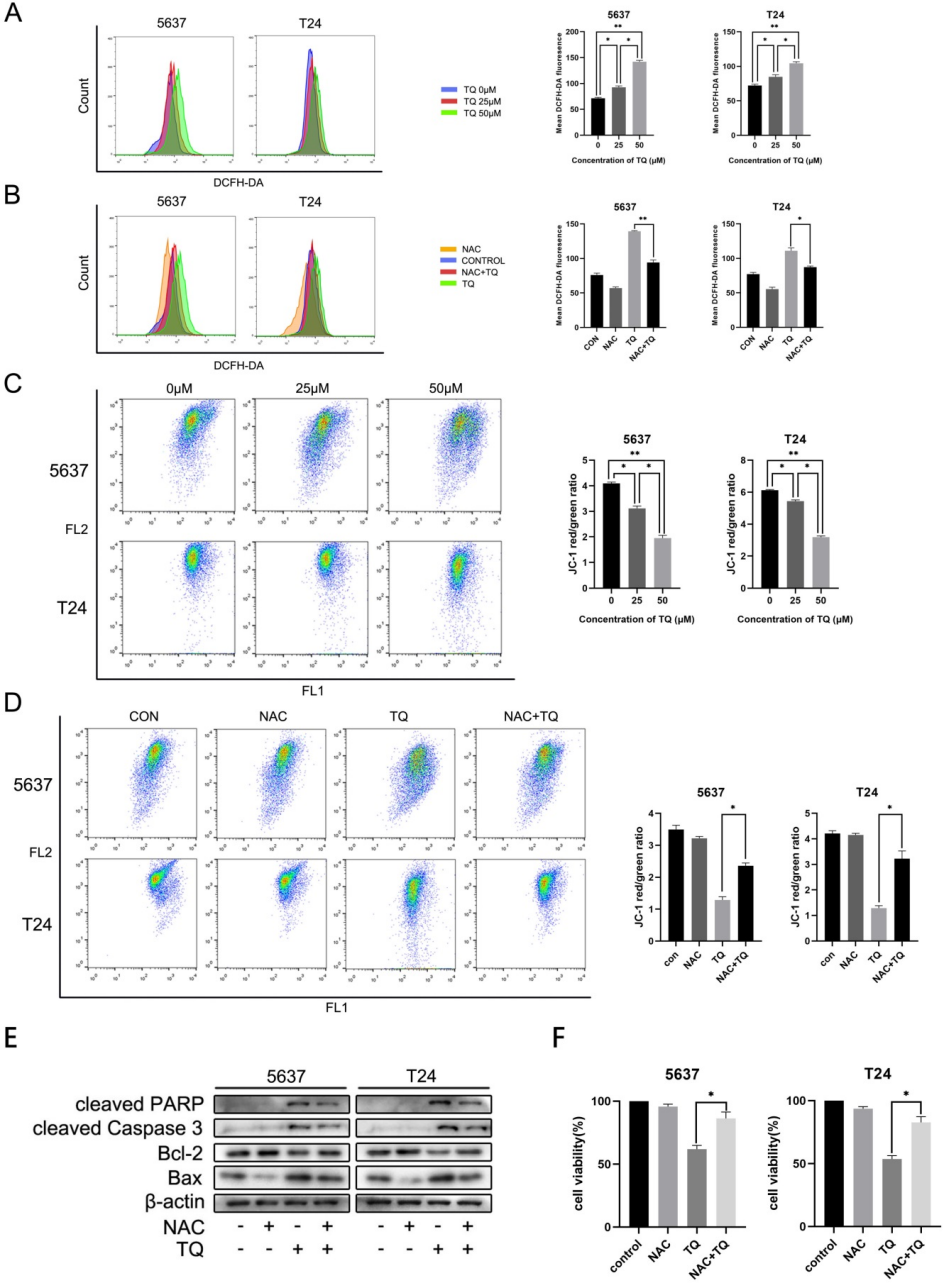
TQ induced ROS activates apoptosis in BC cells. After pretreatment of TQ (0 μM, 25 μM and 50 μM) for 24 h, 5637 and T24 cells were stained with DCFH-DA and JC-1 reagents and then detected with flow cytometry experiment to demonstrate the alterations of intracellular ROS level (**A**) and MMP (**C**), respectively. BC cells were then treated with TQ (50 μM, 24 h) in the presence or absence of NAC (5 mM, 4 h), and again detected to monitor ROS level (**B**) and MMP changes (**D**) using flow cytometry method. WB (**E**) and CCK-8 assays (**F**) were also conducted to test the apoptosis related protein changes and viability status of BC cells, which were same treated as Figure 3B and 3D. MMP: mitochondrial membrane potential; NAC: N-acetyl-L-cysteine; WB: western blot; *p<0.05, **p<0.01.

TQ is reported to activate ROS generation in a range of cancer types 11, 38. To further explore whether TQ could initiate the ROS overexpression in BC cells, DCFH-DA reagent was used to incubate the cells pretreated with TQ (0 µM, 25 µM and 50 µM) for 24 h, and flow cytometry was performed to detect the ROS level. Results showed that intracellular ROS level was dose-dependently upregulated by TQ (Figure 3A). Moreover, the activation effect of TQ on ROS level could be significantly depleted when cells were co-treated with TQ and NAC, a kind of ROS scavenger, compared with the administration of TQ alone (Figure 3B). Due to the tight association between intracellular ROS level and MMP, flow cytometry was also performed to test the alteration of MMP, following the protocols of JC-1 assay. The results demonstrated that MMP was markedly lowered by TQ in a dose-dependent way (Figure 3C). Notably, compared with the administration of TQ alone, the MMP was significantly recovered in the combined treatment of TQ and NAC (Figure 3D), implying that the ROS overexpression is tightly related with damaged mitochondria function.

### TQ upregulated ROS is crucial for TQ induced cell apoptosis in BC cells

Emerging researches have demonstrated that the pro-oxidant effect of TQ is crucial for the anti-cancer potential of TQ 11, 13. With the presence of NAC, TQ upregulated pro-apoptosis proteins were significantly depleted, including Bax, cleaved PARP and cleaved caspase 3. On the contrary, the TQ suppressed anti-apoptotic protein, namely, Bcl-2, was markedly upregulated when cancer cells were co-administrated with TQ and NAC (Figure 3E). CCK8 assay indicated that co-treatment with NAC markedly rescued cell viability in comparison to the TQ treatment alone in both cancer cell lines (Figure 3F). Meanwhile, similar results were also observed in the flow cytometry experiment using FITC and PI staining, suggesting that NAC receded the TQ related cytotoxicity in BC cells (Figure 5F). Collectively, these experiments demonstrate ROS plays a crucial role in TQ induced cell apoptosis.

### TQ initiates impaired autophagic flux in 5637 and T24 cancer cells

It's widely reported that there is a close link between autophagy and apoptosis 25, 27. To investigate whether TQ could induce cell apoptosis via modulating autophagic process, WB assay was performed to detect the expression of autophagy related proteins. ATG7 and Beclin-1 proteins are essential for the formation of autophagosomes and LC3B protein is a widely accepted biomarker for the monitor of autophagic process. The results indicated that a concentration gradient treatment of TQ (0 µM, 25 µM and 50 µM) for 24 h remarkably enhanced the expression of Beclin-1, ATG7 and LC3B proteins (Figure 2H). In general, the downregulation of SQSTM1/p62, a kind of substrate of autophagic degradation, is a clear marker of activated autophagy. However, the enhanced expression of p62 was observed when cells were treated with TQ dose-dependently (Figure 2H), implying that TQ administration impaired the autophagic flux in BC cells.

**Figure 4:**
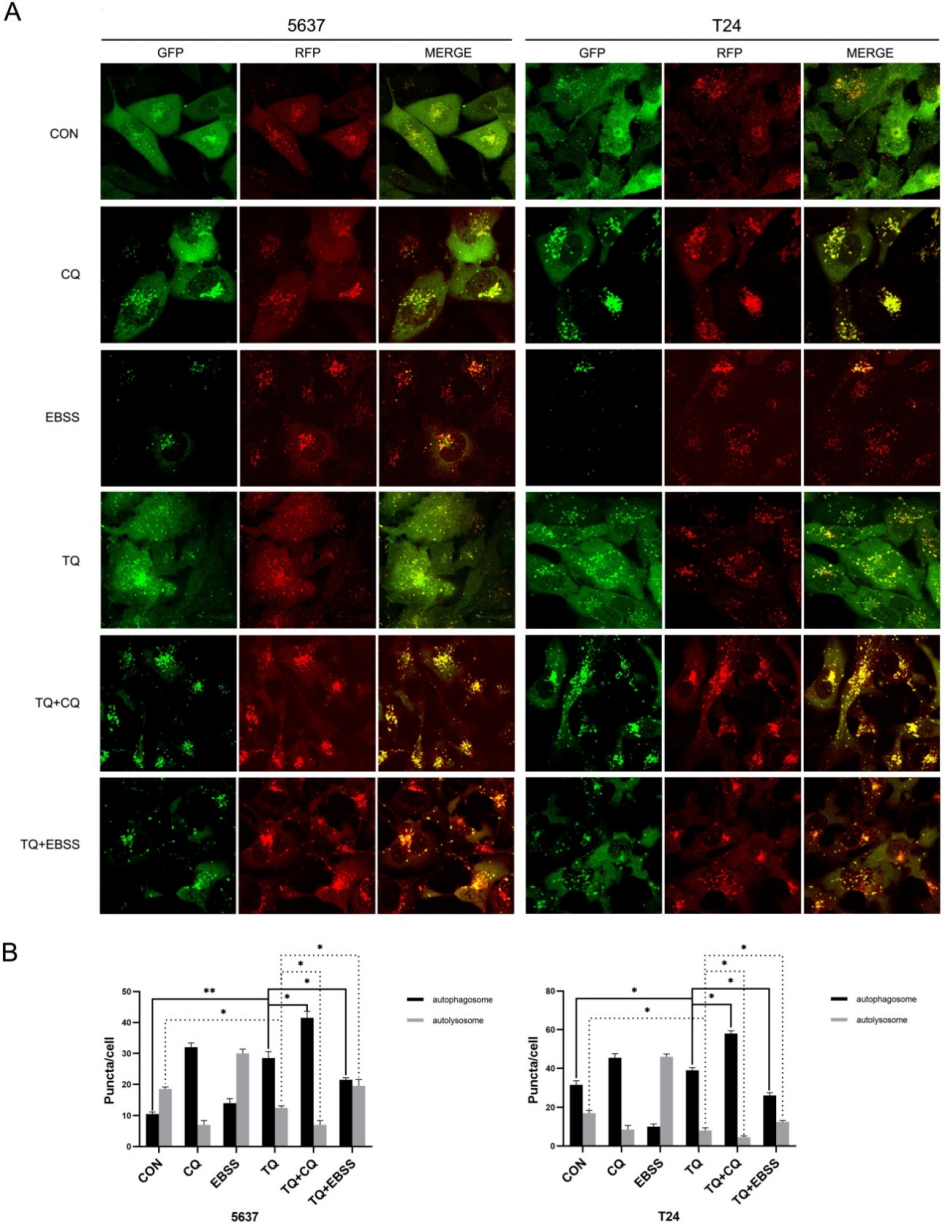
TQ blocks autophagic flux in 5637 and T24 cells. (**A**) 5637 and T24 cell lines stably expressing RFP-GFP-LC3B protein were established using lentivirus transfection, then treated with different administration, including CQ (10 μM, 6 h), EBSS (12 h), TQ (50 μM, 24 h), CQ pretreatment (10 μM, 6 h) with TQ (50 μM, 24 h) and EBSS pretreatment (12 h) with TQ (50 μM, 24 h). Confocal microscope was used to observe the autophagic flux alterations of BC cells under different treatment. (**B**) Quantification analysis of autophagosomes and autolysosomes of cancer cells in Figure 4A. Data are shown as the mean ± standard deviation (SD). CON: control; CQ: Chloroquine; EBSS: Earle's balanced salt solution; *p<0.05, **p<0.01.

To further verify that conclusion, lentivirus carrying RFG-GFP-LC3B (Genechem Co.,Ltd., Shanghai) was used to transfect 5637 and T24 cells. When autophagy is initiated, the LC3B protein is recruited to attach to autophagosomes and presents as yellow puncta (RFP+, GFP+); while when autophagosomes and lysosomes are fused, GFP fluorescence signals are quenched in the acidic autolysosomes environment, presenting as red puncta (RFP+, GFP-). Hence, the alterations of red and yellow signal are used to observe the dynamic state of autophagic flux. As shown in Figure 4A and 4B, TQ treatment (50 µM, 24 h) significantly induced more autophagosomes and less autolysosomes, implying the emergence of impaired autophagic flux. Moreover, Chloroquine (CQ) could block the transformation from autophagosome to autolysosome, and is widely used as an autophagy inhibitor 24. Pretreatment of CQ (10 µM, 6 h) could markedly synergize with TQ, exerting even stronger impairment to autophagic flux of cancer cells (Figure 4A and 4B). Furthermore, cancer cells were cultivated in Earle's balanced salt solution (EBSS) for 12 h to activate the autophagic flux before the TQ administration (50 µM, 24 h), resulting in a remarkable recover of autophagic flux in both cell lines (Figure 4A and 4B).

**Figure 5:**
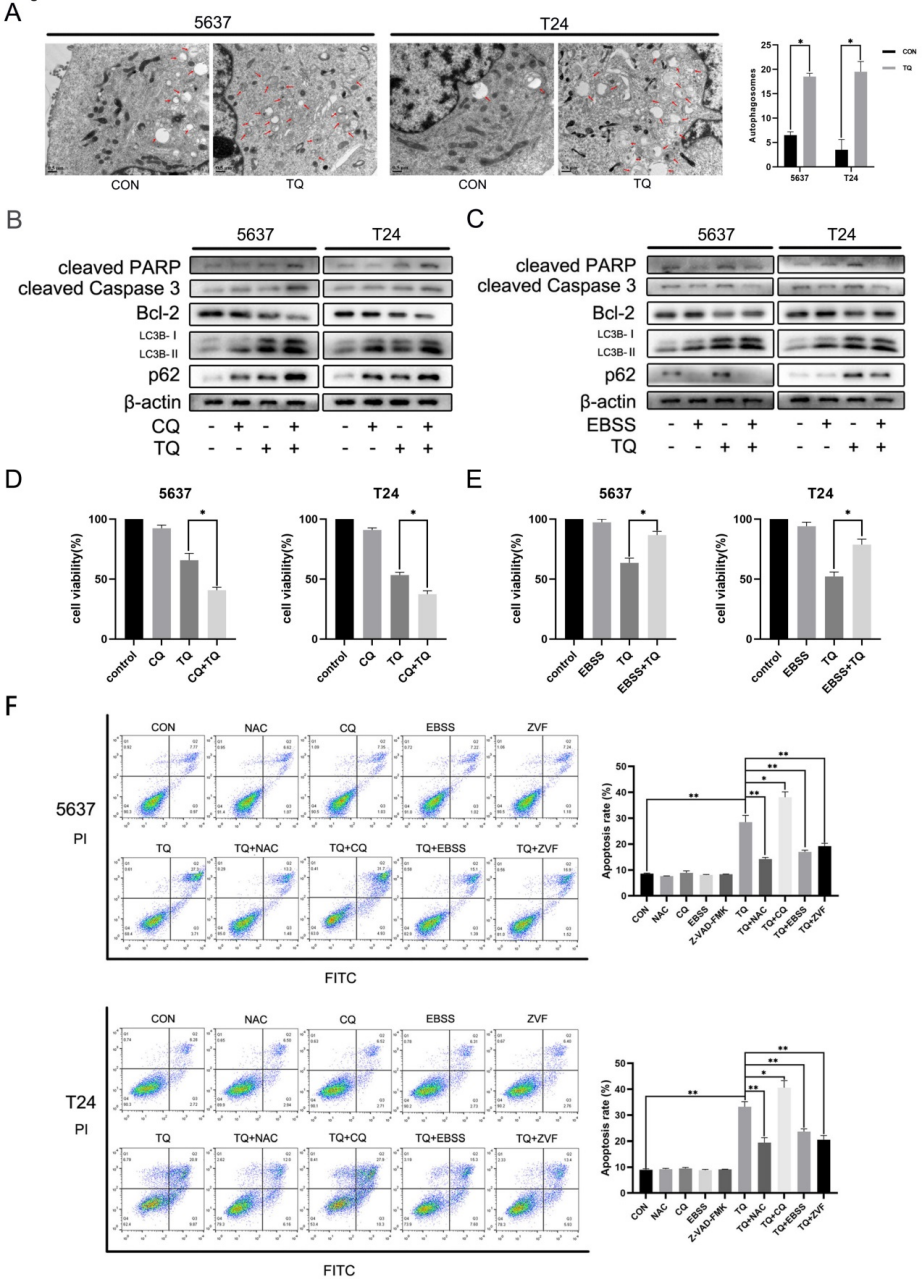
TQ induced Impaired autophagic flux mediates the cell apoptosis of BC. (**A**) 5637 and T24 cells were administrated with TQ of 50 μM for 24 h or not, transmission electronical microscopy was used to observe the autophagosomes formation in two cell lines. (**B, C**) TQ treated (50 μM, 24 h) 5637 and T24 cells were pre-administrated with CQ (10 μM, 6 h) or EBSS (12 h), followed by WB assays detecting the expression levels of apoptosis related proteins (cleaved PARP, cleaved caspase 3 and Bcl-2) and autophagic markers (LC3B and p62). (**D, E**) CCK8 assay was conducted to test the cell viability of BC cells same treated as Figure 5B and 5C. (**F**) BC cells were pre-administrated with control medium or the following administrations: NAC (5 mM, 4 h), CQ (10 μM, 6 h), EBSS (12 h) and ZVF (20 μM), and then exposed to TQ of 50 μM for 24 h. Flow cytometry assay was conducted to test the apoptosis rate of cancer cells co-stained with PI and FITC. ZVF: Z-VAD-FMK; WB: western blot; *p<0.05, **p<0.01.

In addition, after cells were pretreated with TQ (50 µM, 24 h), autophagosomes, characterised with double membrane and intramembranous contents, were markedly upregulated under TEM (Figure 5A), compared to untreated group, further verifying TQ's inhibition on autophagic flux in BC cells.

### TQ induces cell apoptosis via blocked autophagic flux in BC cells

To explore whether TQ induced impaired autophagic flux is involved in the modulation of cell apoptosis, WB experiment, CCK8 and flow cytometry were performed. Firstly, cotreatment with both CQ and EBSS could enhance the expression of LC3B protein compared to treatment of TQ alone, while pretreatment with CQ enhanced the intensity of TQ caused p62 accumulation (Figure 5B), and pretreatment of EBSS reversed the TQ initiated p62 overexpression (Figure 5C), suggesting the enhanced and reduced autophagosomes accumulation, respectively. Furthermore, the CQ induced increased accumulation of autophagosomes, namely further impaired autophagic flux, upregulated the level of cleaved PARP and cleaved caspase 3 proteins and downregulated the Bcl-2 protein (Figure 5B), while the EBSS pretreatment exerted the opposite effect (Figure 5C). In addition, CCK8 assay demonstrated that CQ or EBSS treatment alone will not bring distinct alteration to the viability of both cancer cells, while cotreatment of CQ or EBSS with TQ could significantly reduce (Figure 5D) or recover (Figure 5E) the cell viability, respectively, compared to TQ administration alone. Not surprisingly, after staining with PI and FITC, flow cytometry showed that cotreatment of CQ or EBSS with TQ markedly enhanced or receded the TQ induced apoptosis (Figure 5F), respectively, in consistence with previous results. Taken together, we draw the conclusion that TQ induced impaired autophagic flux plays modulatory role in TQ related cytotoxicity.

### TQ upregulates hsa-miR-877-5p level to reduce PD-L1 expression in 5637 and T24 BC cells

**Figure 6:**
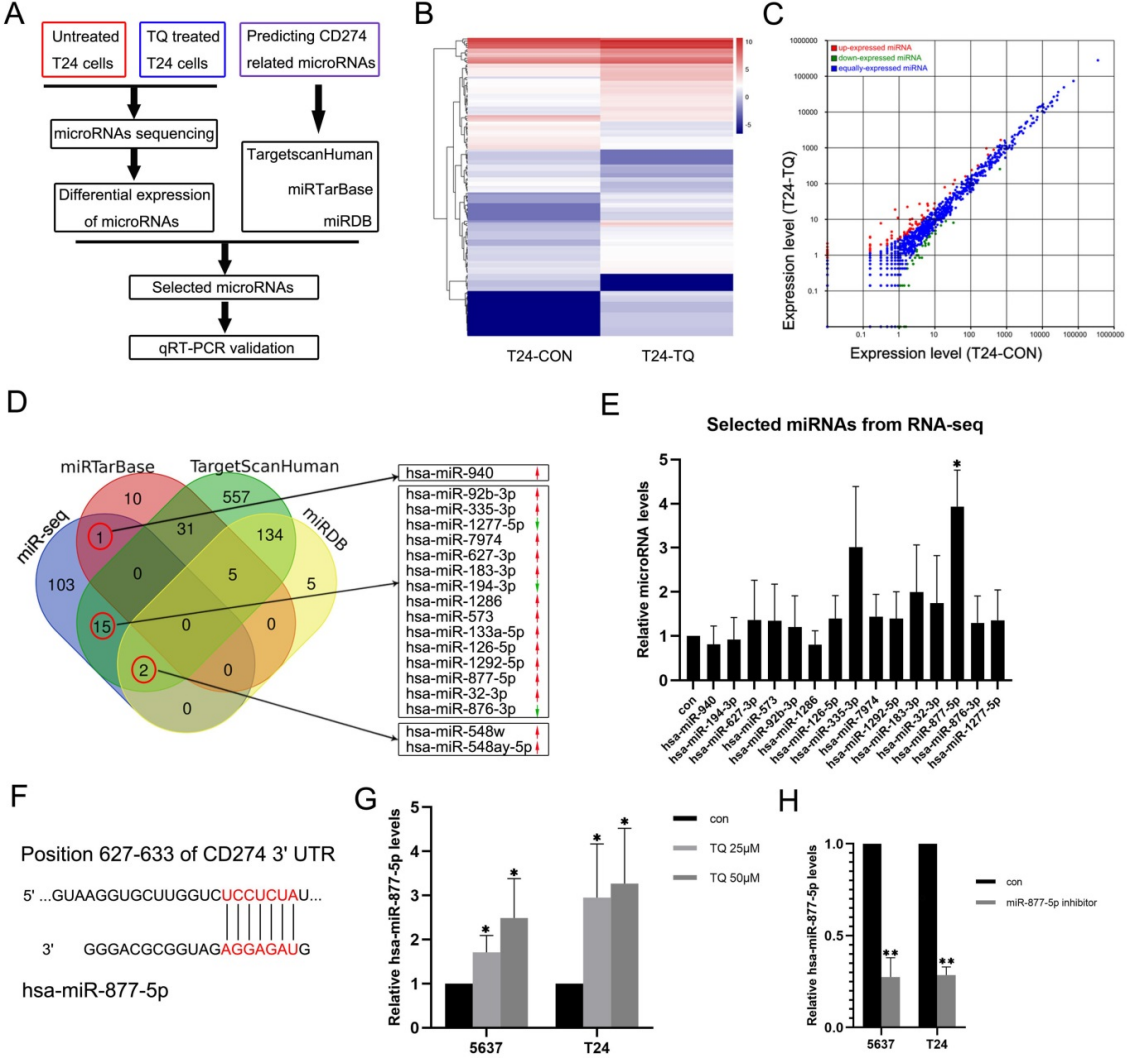
TQ upregulates the miR-877-5p level in BC cells. (**A**) The flow diagram of selecting the potential microRNAs related to the TQ treatment. (**B, C**) Heat map and scatter diagram of microRNA sequencing demonstrating the differential expressions between control and TQ treated group. (**D**) Venn diagram shows the overlapping microRNAs of sequencing results and three online databases (miRTarBase, TargetScanHuman and miRDB), with red arrows representing the relative high expression microRNAs compared to control while green arrows meaning the low expression. (**E**) qRT-PCR validation of selected microRNAs in T24 cells. (**F**) The predicted binding site of miR-877-5p and 3'UTR region of CD274 mRNA. (**G**) qRT-PCR testing the levels of miR-877-5p in TQ treated BC cells. (**H**) T24 and 5637 cells were transfected with inhibitor of miR-877-5p and qRT-PCR was conducted to detect the levels of miR-877-5p. Statistics are exhibited as the mean ± SD of three independent experiments. *p<0.05, **p<0.01.

**Figure 7:**
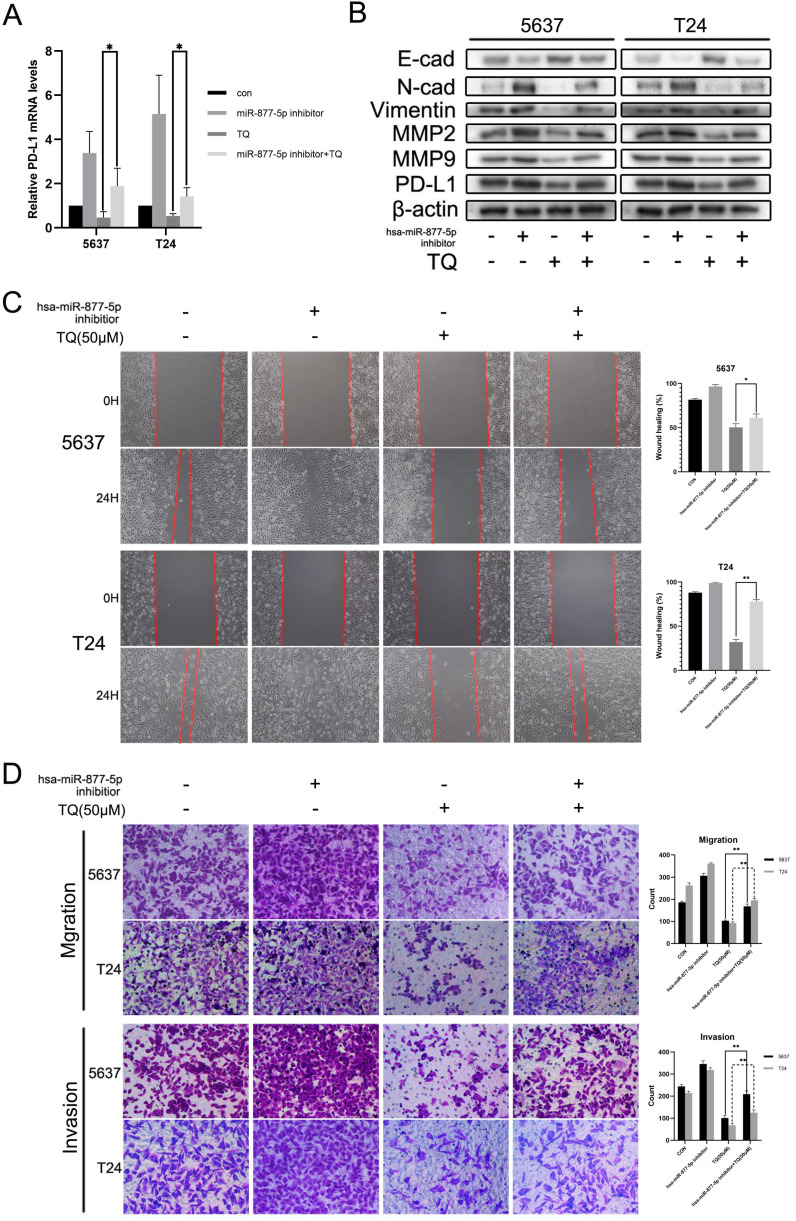
Inhibition of miR-877-5p attenuates the inhibitive role of TQ in migration, invasion and PD-L1 expression of BC cells. (**A**) BC cells were transfected with inhibitor of miR-877-5p, then administrated with TQ (50 μM, 24 h) or not, and qRT-PCR was conducted to monitor the changes of PD-L1 mRNA. (**B**) For 5637 and T24 cells same treated as Figure 7A, expression levels of MMP2, MMP9, E-cad, Vimentin, N-cad and PD-L1 proteins were tested using WB assay. (**C, D**) Cell scratch test, transwell migration and invasion assay of BC cells same treated as Figure 7A. *p<0.05, **p<0.01.

To explore whether TQ could exert its biological effect on tumor via regulating the level of specific microRNA, a microRNA sequencing profile was performed using TQ (25 µM, 24 h) treated T24 cell line with untreated T24 cell line as control group (Figure 6A, B and C, each sample contains three replicates of the same administration). The result demonstrated differential expressions of 121 microRNAs, which were used to overlap with the predicted CD274 related microRNAs obtained from the TargetscanHuman, miRTarBase and miRDB online databases. In the 121 differentially expressed microRNAs, 18 microRNAs were shown to potentially related to the CD274 expression (Figure 6D). Considering the expression level (shown in the results of microRNA sequencing) and the biological functions of these RNAs in previous researchers, qPCR assay was performed to determine the expression levels of 15 microRNAs in TQ-treated (25 µM, 24 h) T24 cells (Figure 6E), and hsa-miR-877-5p was selected as the research target. Besides, with the effect of miR-877-5p inhibitor (Figure 6H), TQ inhibited PD-L1 mRNA level was significantly upregulated (Figure 7A), implying the mediator effect of miR-877-5p in the inhibition of TQ on PD-L1 expression.

### Hsa-miR-877-5p inhibition reverses TQ suppressed PD-L1 expression, as well as the migration and invasion capability of BC cells

Previous researches have indicated the anti-cancer role of miR-877-5p in various cancers 39, 40. To explore whether miR-877-5p can modulate the biological processes of bladder carcinoma, inhibitor of miR-877-5p was used to transfect the BC cells. As presented in Figure 7B, WB assay suggested that when cotreated with TQ, miR-877-5p inhibitor partially recovered the TQ repressed EMT progress, characterized with the enhanced Vimentin and N-cad proteins, as well as the reduced E-cad protein compared with TQ treatment alone. Meanwhile, the inhibitory effect of TQ on MMP2 and MMP9 proteins was also suppressed in the presence of miR-877-5p inhibitor. Furthermore, the similar trend to MMP protein groups was observed in the PD-L1 protein alterations, in accordance with the outcomes of qPCR assay (Figure 7A). Subsequently, wound healing (Figure 7C) and transwell migration assay (Figure 7D) were performed, implying that miR-877-5p inhibitor enhanced the migration ability of BC cells and weakened the inhibitory effect of TQ on the migration of both two cell lines. Moreover, we also conducted the transwell invasion test, verifying the pro-invasion effect of miR-877-5p inhibitor on BC cells, even with the presence of TQ co-treatment (Figure 7D).

### PD-L1 overexpression recedes the repressive effect of TQ on the invasion and migration of 5637 and T24 cells

**Figure 8:**
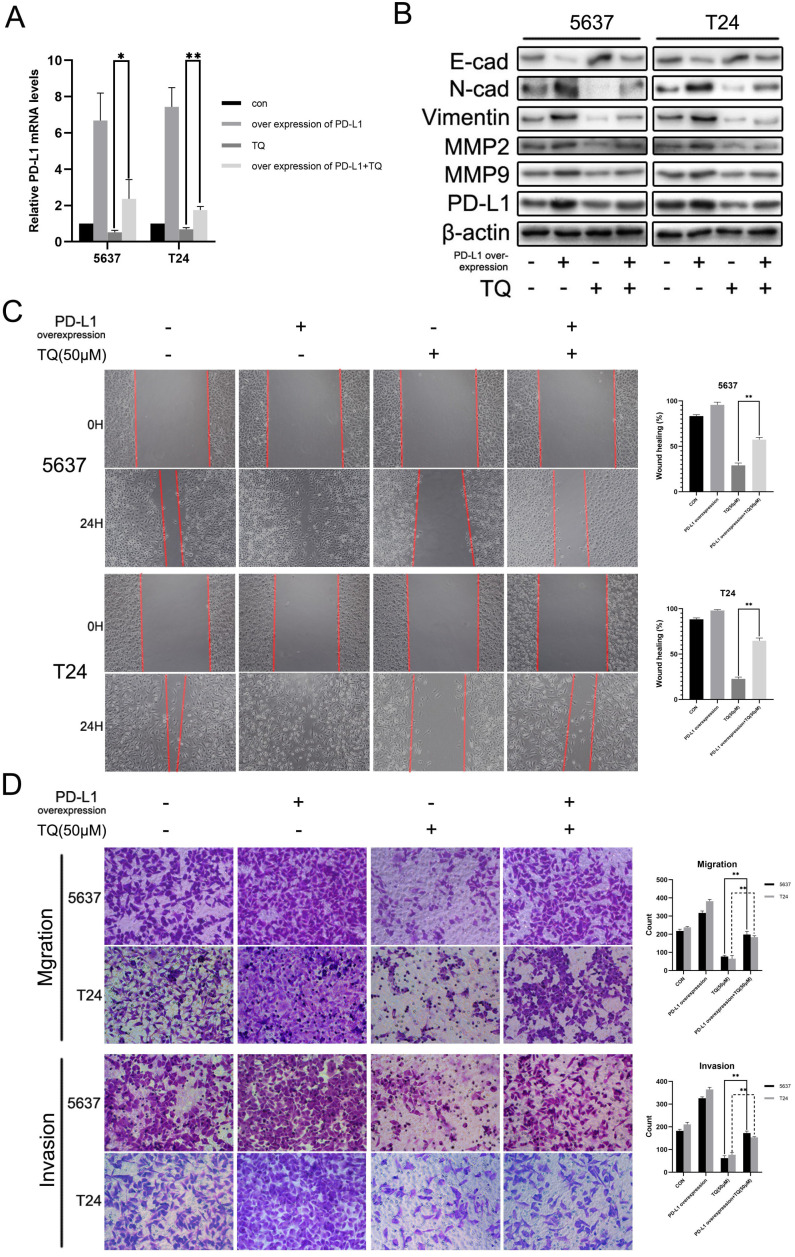
Upregulation of PD-L1 weakens the inhibitive role of TQ in migration, invasion of BC cells. (**A**) 5637 and T24 cells were transfected with lentivirus carrying CD274 transcript, then administrated with TQ (50 μM,24 h) or not, and qRT-PCR was conducted to monitor the level of PD-L1 mRNA. (**B**) For 5637 and T24 cells same treated as Figure 8A, expression levels of MMP2, MMP9, E-cad, Vimentin, N-cad and PD-L1 proteins were tested using WB assay. (**C, D**) Cell scratch test, transwell migration and invasion experiments of 5637 and T24 cells same treated as Figure 8A. WB: western blot; *p<0.05, **p<0.01.

Various researches have demonstrated that PD-L1 could promote the tumor progression through activating the EMT progress and enhancing the MMP group proteins expressions 37, 41, 42. To explore whether PD-L1 could exert the similar effect in BC cells, we transfected 5637 and T24 cancer cells with lentivirus carrying CD274 transcript sequence. As shown in Figure 8A, PD-L1 mRNA level was markedly upregulated and the repressive effect of TQ on PD-L1 was significantly weakened in the presence of PD-L1 overexpression (Figure 8A). Meanwhile, PD-L1 overexpression also contributed to the EMT progress, manifested as the upregulated N-cad and Vimentin proteins with the downregulation of E-cad protein, weakening the inhibitory effect of TQ on EMT progress (Figure 8B). Similarly, PD-L1 overexpression also presented enhanced effect on MMP2 and MMP9 proteins, which also receded the repression of TQ on these two proteins (Figure 8B). In addition, in wound healing and transwell migration assays, PD-L1 overexpression remarkably upregulated the healing rate and the number of migrated cells in two cell lines (Figure 8C and D). As expected, the TQ repressed migration ability was markedly recovered due to the overexpression of PD-L1 (Figure 8C and D). In addition, transwell invasion assay also suggested that PD-L1 overexpression notably enhanced the invasion ability and reversed TQ inhibited invasion of both 5637 and T24 cells.

### TQ refrains the growth and metastasis of bladder carcinoma *in vivo*

**Figure 9:**
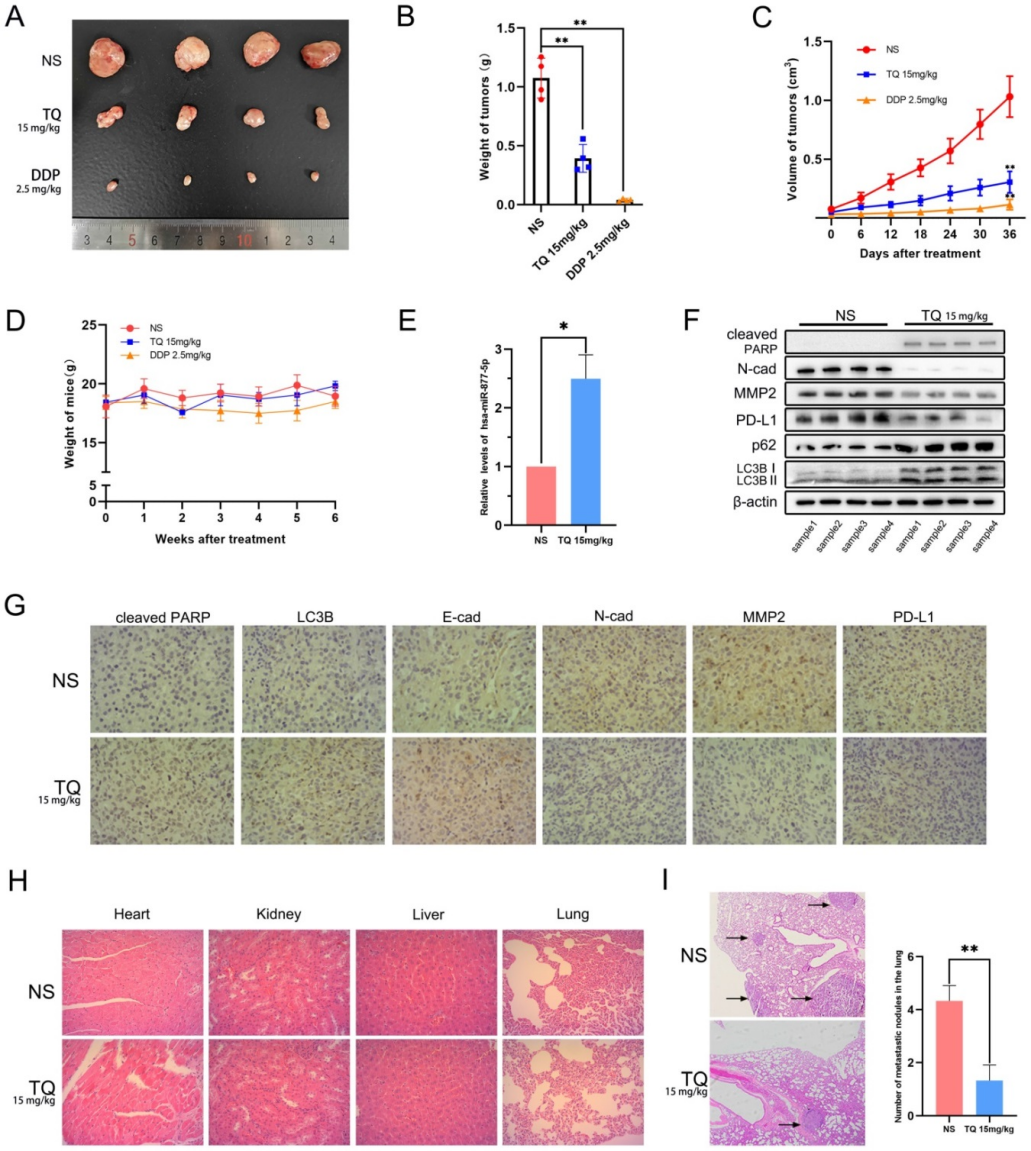
The anti-tumor effect of TQ is proved in animal model of bladder carcinoma, which was established by injection of T24 cells subcutaneously or through the tail veins. (**A**) Gross samples of bladder tumors with different treatment. (**B, C**) Subcutaneous tumors were weighed and the volume changes of tumors were recorded and calculated. (**D**) Body weight measurement presented no significant variations between three groups. (**E**) qRT-PCR experiment was conducted to test the level of hsa-miR-877-5p in tumor tissues. (**F**) WB experiment was used to detect the alterations of N-cad, MMP2, PD-L1, cleaved PARP, p62 and LC3B proteins in tumor tissues. (**G**) For nude mice treated with NS and TQ administration, immunochemistry experiment was also performed to test the variations of different proteins in xenograft tumors, including cleaved PARP, LC3B, MMP2, PD-L1, E-cad and N-cad. (**H**) H&E staining was used to test the organ related toxicity in groups of NS and TQ administration. (**I**) The metastatic nodules in mice lungs with NS and TQ administration were observed and counted by H&E staining. NS: normal saline; DDP: cisplatin; WB western blot; *p<0.05, **p<0.01.

To detect whether TQ could exert its cytotoxicity effect *in vivo*, a subcutaneous tumor xenograft model was established in nude mice using T24 cells. Results showed that both TQ (15 mg/kg) and DDP (2.5 mg/kg) administration could remarkably lighten the weight (Figure 9A and B) and shrink the volume of bladder tumors (Figure 9C) without causing significant body weight alterations of nude mice (Figure 9D). As expected, TQ administration significantly upregulated the level of miR-877-5p in tumor tissue compared to NS-treated group (Figure 9E). Furthermore, WB (Figure 9F) and immunohistochemical assays (Figure 9G) of the tumor tissue indicated that TQ treatment also upregulated the level of cleaved PARP, E-cad, LC3B and p62 proteins, in the meantime reduced the expression of N-cad, MMP2 and PD-L1 proteins. However, no significant toxicity was found in the major organs of TQ treated nude mice (Figure 9H). Moreover, in the lung metastatic model, TQ administration was also found to remarkedly reduce the number of metastatic nodules of bladder carcinoma (Figure 9I). Collectively, it could be concluded that TQ can inhibit both growth and metastasis of bladder carcinoma *in vivo*.

## Discussion

As the 10^th^ most common cancer type globally 1, 2, bladder carcinoma causes nearly 170000 deaths annually^9^. Besides, approximately 573000 new cases of bladder carcinoma are diagnosed every year 2. In terms of treatment, the requirement of close follow-up and multiple endoscopic therapies has made bladder carcinoma among the most expensive cancers to care for a personal patient 43, bringing large social and economic burden to the world. Despite the great advances in the systemic treatment of bladder carcinoma in latest years 6, 7, bladder carcinoma patients still suffer high possibility of tumor progression. Nearly 15%-20% of NMIBC cases will turn into MIBC diseases 44, and for MIBC patients, nearly 50% of them will ultimately suffer distant metastasis even after the RC and pelvic lymph nodes dissection 9, 45. Therefore, developing novel treatment method of bladder carcinoma is still of great significance.

TQ attracted increasing attentions of scientists because of its abundant biological functions. The crucial mechanism mediating the diverse bioactive properties lies in the lipophilic quinine constituent in its chemical structure 17, which facilitates the access of TQ to the cellular and subcellular units as well as the interaction between TQ and intracellular transcription factors or kinases 46, 47, endowing TQ excellent potency in the treatment of various illness, cancer included 11-13. Therefore, we conducted this research to exploit whether TQ could suppress the tumorgenesis of bladder carcinoma.

Apoptosis, one of the programmed cell death mechanisms 48, is under accurate control by multiple signaling pathways and is essential for the homeostasis of single cell and organ 49. This suicidal process could eliminate superfluous or damaged cells, maintaining the subtle equilibrium between cell proliferation and cell death 50. One of the most distinct characteristics of malignant cells is the ability to avert the apoptosis process, making it possible to permanently proliferate for single cancer cell 51. Hence, triggering apoptosis or targeting apoptosis related proteins have become potential methods of developing novel treatment to various malignancies 52, 53. Our research showed that TQ administration concentration-dependently upregulated the apoptosis rate of cancer cells by increasing the expression level of cleaved PARP, Bax and cleaved caspase 3 proteins while decreasing the expression level of anti-apoptotic proteins, such as Bcl-xl and Bcl-2 proteins. Moreover, ZVF, an antagonist of cleaved caspase 3, could reverse the above alteration induced by TQ, suggesting the critical role of cleaved caspase 3 in TQ triggered apoptosis in cancer cells.

ROS could mediate the TQ activated apoptosis in cancer cells. It is widely acknowledged that ROS exerts pivotal effects in the equilibrium of cellular energy, and alteration of the delicate balance is one of the characteristics of carcinogenesis 21, 54. Targeting the ROS in cancer cells has become one of the most effective strategies to combat cancer 20, 55, 56, as overproduction of ROS could inflict damage to cancer cell via multiple methods 57. Firstly, ROS induces the mutations of cell DNA, causing damages to multiple components of DNA through the reactive chemical property 58; secondly, ROS disturbs the normal protein functions by ROS-mediated cleavage of peptide and the nitrosylation of proteins, resulting in the dysfunction of related proteins 59, 60; thirdly, ROS attacks the lipid membrane of organells by lipid peroxidation, causing substantial damage 61. In addition, excessive ROS damages the membrane of mitochondria, decreasing the MMP and resulting in the release of cytochrome C, which initiates the apoptosis cascades 22, 57, 62. In this study, TQ was proved to dose-dependently induce upregulation of ROS and downregulation of MMP in BC cells. Furthermore, the role of TQ in ROS was neutralized by NAC, a kind of ROS scavenger. Meanwhile, TQ induced apoptosis was also rescued by NAC, implying the crucial role of ROS in TQ activated apoptosis in BC cells.

Impaired autophagic flux also facilitates TQ induced cells apoptosis. Autophagy is a multi-stage biological procedure, involving the participation of lysosome, mediating materials degradation, nutrient recycling and metabolic adaptation 25, 27. The intact, dynamic and continuous procedure of autophagy is called autophagic flux, involved in various biological procedures, cancer cell apoptosis included 24. Although moderate autophagy is considered to ensure the cancer cell survival in the carcinogenesis, various stressors induced complete and prolonged autophagy will deplete the biomaterials of cancer cell, ultimately resulting in the apoptosis 25, 27, 28, 63. In addition, the blocked autophagic flux was reported to cause the accumulation of autophagosomes, which could also initiate the apoptosis process. For examples, paroxetine promotes lung cancer cells apoptosis through blockage of autophagic flux 64, and dipyridamole elicits the anti-proliferative effect on prostate cancer cells via impairing the autophagic flux 65. In this research, TQ blocked the autophagic flux of 5637 and T24 cancer cells, leading to the accumulation of autophagosomes, which could be enhanced or attenuated by CQ or EBSS pretreatment, respectively. And the alteration of apoptosis process is in consistence with the changes of autophagosomes, suggesting the assistant effect of impaired autophagic flux in the TQ exerted anti-cancer property. Besides, it still needs further exploration whether intact autophagy process could influence the cancer progression.

MicroRNA has been widely reported as the crucial regulator in multiple biological processes 29, 30. Through microRNA sequencing, screening the online database and qPCR verification, we identified hsa-miR-877-5p as the research objective. According to previous researches, miR-877-5p generally works as a cancer suppressor in multiple malignancies. It is found that miR-877-5p inhibits the cell proliferation, migration and invasion of hepatoma 40 and suppresses the cell growth of gastric carcinoma 39. Also, more researches demonstrate that various long non-coding RNAs or circular RNAs facilitate the tumorgenesis of different carcinomas by decreasing the level of miR-877-5p 66-70, further verifying the suppressive effect of miR-877-5p in cancer development. In accordance with previous researches, the results of our experiments showed that TQ significantly upregulated the miR-877-5p expression level, and the inhibitor of miR-877-5p markedly upregulated the mRNA levels of CD274 (PD-L1), suggesting that CD274 mRNA is the downstream target of miR-877-5p. Furthermore, the miR-877-5p inhibitor remarkably enhanced the TQ attenuated EMT progress, invasion and migration of bladder carcinoma, confirming the regulative effect of miR-877-5p on TQ induced anti-cancer bioactivity.

TQ restrained PD-L1 also exerts a crucial effect on TQ inhibited EMT progress, migration and invasion of BC cells. Recently, a growing number of researches indicate that, beyond immune evasion, PD-L1 also participates the intrinsic signaling pathways and enhances the stemness, metastasis or even chemoresistance of cancer cells 71. For examples, PD-L1 initiates the PI3K/AKT axis to accelerate the progression of breast carcinoma 72; EMT and invasion are enhanced via PD-L1 initiated RAS/ERK/EMT axis in glioblastoma 73; and in renal cancer cells, EMT is also promoted by PD-L1 through activating SREBP-1c 74. Similarly, the outcomes of our study indicated that TQ significantly decreased the expression of PD-L1 and overexpression of PD-L1 markedly heightened the TQ repressed EMT progress, migration and invasion of BC cells, in consistence with previous reports, indicating the crucial effect of PD-L1 in TQ's anti-cancer effect. Interestingly, recent researches also reported that EMT process enhanced PD-L1 expression, and the subsequent immune escape of cancer cell 34, 75, implying the potential bilateral regulation mechanism between the EMT process and PD-L1 protein 34, 35, which requires further exploration in the future.

Taken together, this study demonstrates that TQ could induce the overproduction of ROS and downregulation of MMP, as well as the impaired autophagic flux, to initiate the apoptosis of BC cells. As well, we prove TQ activates miR-877-5p/PD-L1 axis to inhibit the EMT procedure and invasion of BC cells, hence further inhibits the progression of bladder carcinoma. It can be concluded that, as a multi-effective phytomedicine discovered for decades, TQ could be used as a novel treatment strategy, or play an adjuvant role in the systemic therapy of human bladder carcinoma.

## Figures and Tables

**Figure 10:**
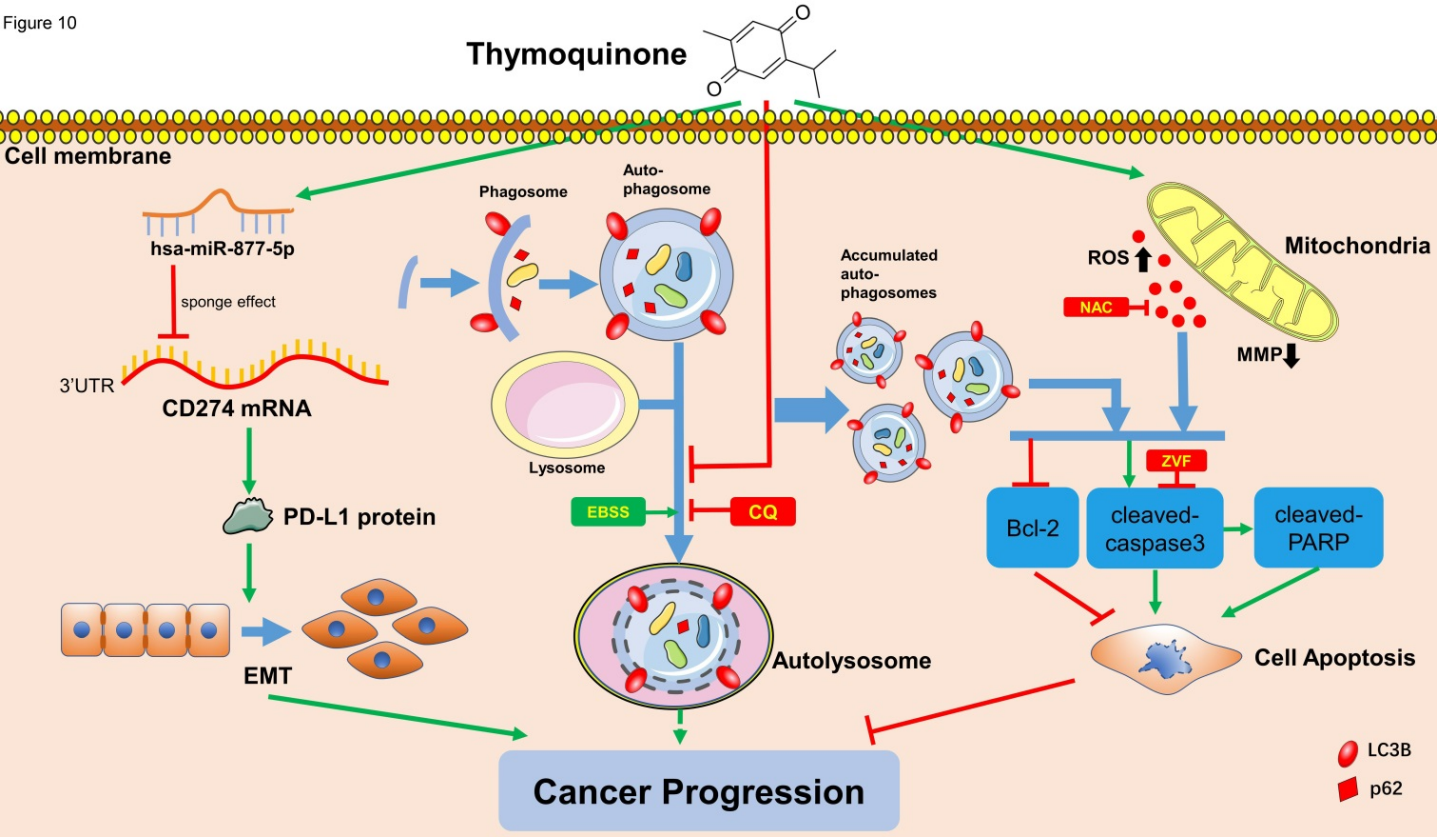
Schematic of the proposed mechanism in this study. On one hand, TQ induces the overproduction of the intracellular ROS, which is closely associated with the mitochondrial dysfunction and MMP decrease. On the other hand, TQ blocks the cellular autophagic flux, resulting in the accumulation of autophagosomes. Together with the upregulated ROS, impaired autophagic flux activates the caspase dependent apoptosis, inhibiting the cancer progression. Nevertheless, whether intrinsic autophagic flux facilitates the cancer cells survival and cancer progression depends on the cell type and extent of activated autophagy, requiring further exploration. In addition, TQ upregulates the levels of hsa-miR-877-5p in 5637 and T24 cells, which decreases the level of PD-L1 protein, resulting in the attenuation of migration and invasion ability of BC cells. Arrows connecting different items present the inhibiting (red) or promoting (green) effect of various factors.

## Abbreviations

TQ: thymoquinone
BC: bladder carcinoma
RC: radical cystectomy
ROS: reactive oxygen specifics
NAC: N-Acety-L-Cysteine
EMT: epithelial mesenchymal transformation
PD-L1: programmed death ligand 1
MMP: mitochondrial membrane potential
SDS-PAGE: dodecyl sulfate sodium salt-polyacrylamide gel electrophoresis
PVDF: Polyvinylidene Fluoride
RT: room temperature
TBST: Tris-buffered saline Tween
MMP: Mitochondrial membrane potential
CQ: chloroquine
EBSS: Earle's balanced salt solution
h: hour
CON: control
ZVF: Z-VAD-FMK
WB: western blot
NS: normal saline
DDP: cisplatin
